# Immunoprognostic analysis of indoleamine 2,3-dioxygenase 1 in patients with cervical cancer

**DOI:** 10.1097/MD.0000000000039733

**Published:** 2024-09-20

**Authors:** Cong Xu, Min Wang, Chaowen Chen, Yonghong Xu, Fang Liu, Guangming Wang

**Affiliations:** a School of Clinical Medicine, Dali University, Dali, Yunnan, People’s Republic of China; b Chinese People’s Life Safety Research Institute, Huaxi Hospital, Sichuan University, Chengdu, Sichuan, People’s Republic of China; c Department of General Surgery, Banan Hospital Affiliated to Chongqing Medical University, Banan, Chongqing, People’s Republic of China; d Center of Genetic Testing, The First Affiliated Hospital of Dali University, Dali, People’s Republic of China.

**Keywords:** cancer treatment, cervical cancer, immune infiltration, indoleamine 2,3-dioxygenase 1, prognosis

## Abstract

The incidence of cervical cancer is increasing. Immunotherapies show better patient outcomes than monotherapies; however, the mainstay treatment for cervical cancer remains surgery and chemotherapy. Indoleamine 2,3-dioxygenase 1 (IDO1) acts on multiple tryptophan substrates, exhibiting antitumor, immunomodulatory, and antioxidant activities. Despite the association of elevated IDO1 expression with unfavorable outcomes in various cancers, its precise function in cervical cancer remains ambiguous. Here, we explored the prognostic significance of IDO1 in cervical carcinoma. Gene expression datasets were obtained from The Cancer Genome Atlas. Gene Expression Omnibus datasets were used for differential expression and functional correlation analyses. Using Human Protein Atlas alongside Tumor-Immune System Interaction Database, we assessed the association of IDO1 with survival rates. Given the link between cervical cancer prognosis and immune invasion, CIBERSORT was used to assess the connection between immune cells and IDO1, while the percentage of tumor-penetrating immune cells based on IDO1 expression in cervical cancer patients was analyzed using Tumor-Immune System Interaction Database. Incorporating a clinicopathological characteristic-based risk score model with IDO1 risk score, we devised a nomogram to predict cervical cancer patient survival. The effects of IDO1 in immune regulation and its prognostic significance were validated using data from patients with cervical cancer obtained from The Cancer Imaging Archive database. Compared with that in normal cervical tissues, IDO1 expression was significantly upregulated in cervical cancer tissues and significantly correlated with cervical cancer progression and prognosis. IDO1 expression showed a positive association with monocyte and macrophage abundance, while exhibiting a negative correlation with that of endothelial cells and eosinophils. Cox regression analyses highlighted IDO1 as the core immune gene implicated in cervical cancer. Gene Ontology and Kyoto Encyclopedia of Genes and Genomes analyses revealed an association of IDO1 with the metabolic pathways of tryptophan, phenylalanine, and tyrosine. Univariate and multivariate analyses revealed that elevated IDO1 expression correlates markedly with cervical cancer outcomes, suggesting it as a promising therapeutic target. The Cancer Imaging Archive data analysis revealed that the impact of anti-PD1 and CTLA4 therapy is more pronounced in cervical cancer patients exhibiting elevated IDO1 expression. IDO1 is a potential target for immunotherapy for cervical cancer.

## 1. Introduction

Cervical cancer is a common gynecological malignant tumor and the fourth most common cause of cancer-related death worldwide, with an incidence of 1.39% and a mortality rate of 0.82% in 2020. In 2020 alone, an estimated 604,000 new cases of cervical cancer and 342,000 related deaths were reported worldwide.^[1]^ Among the 53 countries included in the analysis of mortality trends over the past 5 years, mortality rates have increased in 5 countries.^[2]^ More specifically, the incidence rates have increased, the age of onset has gradually decreased, and mortality from early-onset cervical cancer has increased in recent generations.^[1]^ Studies have predicted that by 2040, there will be 855,130 novel cases of cervical cancer and 533,588 related deaths worldwide, posing a huge economic and health burden globally.^[3]^ Global population growth has markedly contributed to the absolute burden of cervical cancer.^[3]^

Tumor prognosis has been shown to correlate with changes in the number of tumor-infiltrating lymphocytes and specific immune subsets.^[4]^ Mainstay treatments for cervical cancer include surgery plus adjuvant chemoradiotherapy or chemoradiotherapy alone. Antibodies that target immune checkpoint proteins, including cytotoxic T lymphocyte antigen-4 (CTLA4), programmed cell death protein 1 (PD1), and PD-L1, have immensely improved treatment outcomes and patient survival rates across a broad spectrum of tumors. Notably, strategies combining surgery with immunotherapy result in better patient outcomes than monotherapies.^[5]^

Indoleamine 2,3-dioxygenase 1 (IDO1) catalyzes the production of n-formylkynuridine from tryptophan. It is involved in purine production, and its overexpression leads to the depletion of tryptophan, thereby reducing cell energy levels, which prevents dendritic cell (DC) maturation and thus causes proinflammatory T-cell apoptosis. Moreover, IDO1 inhibits the function of T lymphocytes, as tryptophan catabolites exert cytotoxic effects on NK and T-cells.^[6,7]^

Numerous tumor types upregulate IDO1 to expedite tryptophan breakdown and establish an immunosuppressive milieu. IDO1 upregulation or elevated levels of renal purines are associated with poor patient prognosis and resistance to immune checkpoint therapy.^[8]^ IDO1 is upregulated in a variety of cancer and immune cells within the tumor microenvironment. Further, high IDO1 expression has been associated with the occurrence of metastatic pancreatic ductal adenocarcinoma, acute myeloid leukemia, as well as rectal, ovarian, endometrial, lung, and other cancers.^[9]^ Nonetheless, the precise function of IDO1 within the context of cervical cancer remains elusive.

In this study, we sought to elucidate the intricate association between IDO1 expression, immune infiltration, and prognosis in cervical cancer, with the ultimate goal of identifying novel therapeutic targets. This manuscript was written following the STREGA checklist.

## 2. Methods

### 2.1. Cervical cancer data

Gene expression data and clinical details for cervical cancer cases were obtained from The Cancer Genome Atlas (TCGA, accessible at https://portal.gdc.cancer.gov/). mRNA expression and clinical data were combined and organized using Perl (v5.30.3).

### 2.2. Validation using the Gene Expression Omnibus (GEO) dataset and Human Protein Atlas (HPA)

To assess the differential expression of IDO1 in cervical cancer, the GSE7410, GSE29570, GSE39001, and GSE63514 datasets were obtained from the GEO database, with GSE39001 specifically featuring microarray analyses of both healthy individuals (n = 12) and cervical cancer patients (n = 43). The GSE7410 dataset encompasses raw data on gene expression profiles from healthy individuals (n = 5) and patients with cervical cancer (n = 40). The GSE29570 dataset includes unprocessed transcript expression data from both healthy individuals (n = 17) and patients with cervical cancer (n = 45). The GSE63514 dataset contains raw transcript expression data from healthy donors (n = 24) and patients with cervical cancer (n = 104). Appropriate statistical methods were selected using R (4.2.1) (stats and car packages). Data visualization was performed using the ggplot2 software. The Wilcoxon rank-sum test was utilized to evaluate the variance in IDO1 expression between cervical cancer tissues and healthy tissue, with *P* < .05 indicating significance.

### 2.3. Receiver operating characteristic (ROC) curve analysis

Upon inputting the gene identifier “IDO1” into the Tumor-Immune System Interaction Database (TISIDB; accessible via http://cis.hku.hk/TISIDB), we conducted an analysis to discern correlations between patient cohorts at a high and low risk. The association between the Kaplan–Meier curve representation and overall survival (OS) was obtained.

We used multivariate and multifactorial Cox regression analyses to determine whether IDO1 is a standalone prognostic factor for cervical cancer and an independent risk factor. Utilizing the survival and limma packages in R, a detailed examination was performed to identify the relationship between patient OS rates based on IDO expression. In total, 306 cervical cancer and 4 normal samples were analyzed using data from TCGA database. The area under the ROC curve (AUC) was calculated and depicted using the pROC package in R. The ggplot2 package was used to evaluate the prognostic and diagnostic merit of IDO1.

### 2.4. Examining the relationship between IDO1 expression and immune cell distribution

The TIMER2.0 platform (http://timer.cistrome.org/) was employed to analyze a comprehensive dataset of tumor transcriptomes along with immune cell infiltration metrics, with the goal to evaluate the composition of immune cells infiltrating tumors. Briefly, 20 immune cell types, including CD8 + T-cells, CD4 + T-cells, regulatory T-cells (Tregs), B-cells, neutrophils, monocytes, macrophages, DCs, NK cells, mast cells, cancer-associated fibroblasts, common myeloid progenitors, common lymphoid progenitors, endothelial cells, eosinophils, granulocyte-monocyte progenitors, hematopoietic stem cells, follicular helper T-cells, γδ T-cells, NK T-cells, and myeloid-derived suppressor cells, were analyzed. Their association with the IDO1 expression was visualized. Scatter plots were used to illustrate the correlation between gene expression and immune infiltration.

### 2.5. Visualization of immune cell infiltration

We employed the corrplot and CIBERSORT tools available in R (4.3.1) for the analysis of RNA-sequencing data from individuals diagnosed with cervical cancer, extracting immune cells associated with cervical cancer from the original data file and visualizing the correlation between 20 infiltrating immune cell types in the heat map.

### 2.6. Correlation of the expression of IDO1 and immunomodulatory factors

Correlation of immune stimulators and inhibitors to IDO1 expression in cervical cancer patients using The TISIDB (*P* < .05). The selected immunostimulatory factors were ICOS, C10orf54, CD27, CD28, CD40LG, CD48, CD70, CD86, CD276, CXCL12, and ICOSLG, whereas the selected immunosuppressive factors were ADORA2A, BTLA, CD96, CD244, CD160, CD274, CSF1R, IL10, and IL10RB

### 2.7. Gene Ontology (GO) and Kyoto Encyclopedia of Genes and Genomes (KEGG) enrichment analyses

To assess the functional significance of IDO1 expression, we performed GO and KEGG enrichment analyses and presented the results using ggplot2 in R (3.3.6). A protein–protein interaction (PPI) network between immunosuppressants and immunostimulators associated with IDO1 was established using the STRING website (https://cn.string-116 db.org/).

### 2.8. Correlation among immune genes

The reshape2, ggplot2, ggpubr, and corrplot packages in R were used to visualize the correlation between immune factors associated with IDO1 and the relationship between IDO1 and immune modulators. The ggpubr package was used to perform Spearman correlation analysis of infiltrating immune cells with diagnostic markers, and the findings visualized through the “ggplot2” package.^[10]^

### 2.9. Construction of an independent prognostic model

To screen for immune genes linked to cervical cancer prognosis, we utilized the survival and limma tools in R and Cox regression analysis. An immune prognosis model related to IDO1 was established and visualized using a forest plot. To identify immune genes that influence cervical cancer prognosis, we utilized the survival and limma packages in R along with Cox regression analysis. Variables other than the risk score, such as age, sex, grade, and TMN stage, were also analyzed.

The pheatmap package in R was used to calculate the risk score of cervical cancer samples in the public database, and samples were separated into high- and low-risk groups in line with the median risk score. R was used to visualize the risk curves, survival status maps, and heat maps of immunosuppressants and immunostimulants.

### 2.10. Immune gene risk score and construction of a nomogram

To assess the predictive capability of the risk score for patient outcomes, both univariate and multivariate Cox regression models were applied. Furthermore, by employing the “survival” package in R, we produced survival and time-variant ROC curves to determine the predictive precision of the risk score.

A nomogram was constructed by combining the cervical cancer risk score, clinical grade, and stage (T, N, and M). The rms package was used to predict the 1-, 2-, and 3-year OS of patients with cervical cancer. The creation of a calibration curve led to the determination of the prediction’s precision in the column graph, achieved via comparative analysis.

### 2.11. Immunotherapy analysis

The data file of the *IDO1* gene immunity score in cervical cancer was acquired from The Cancer Imaging Archive database (http://tcia.at/). In R, the limma and ggpubr packages facilitated the merging and processing of the score, along with the IDO1 expression file, to display the outcomes.

### 2.12. Statistical analysis

R language 4.3.1 (https://www.r-project.org/) and several publicly available packages (R x64 3.5.1) were used for statistical analysis. Comparisons between 2 groups were conducted using the *t* test. Survival analysis was conducted using the Cox proportional hazards model. The genetic factors linked to cervical cancer prognosis were examined using Cox regression analysis. A *P*-value <.05 was indicative of statistical significance.

## 3. Results

### 3.1. Validation of IDO1 expression in cervical cancer using the HPA database

**Figure 1.**
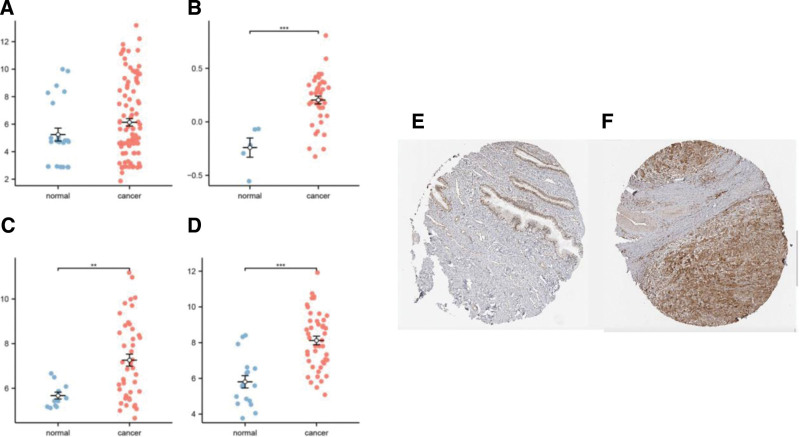
IDO1 is differentially expressed in cervical cancer and normal tissues in GEO datasets. (A) IDO1 expression in GSE63514. (B) IDO1 expression in GSE2950. (C) IDO1 expression in GSE39001. (D) IDO1 expression in GSE7410. (E) Protein expression levels of IDO1 in normal cervical tissues in the Human Protein Atlas database. (F) Protein expression levels of IDO1 in cervical cancer tissues in the Human Protein Atlas database.

Clinical and mRNA expression data of cervical cancer patients and healthy donors, obtained from TCGA database, were examined and confirmed by ensuring data integrity and consistency and assessing quality and metrics. To further demonstrate the characteristics and potential causes of differential IDO1 expression between patients with cervical cancer and healthy donors, we analyzed transcriptomics data from the GSE7410, GSE29570, GSE39001, and GSE63514 datasets. Our analysis revealed a significant upregulation of IDO1 in cervical carcinoma specimens when compared with healthy tissue across all examined datasets (*P* < .01; Fig. 1A–D).

HPA data revealed the localization of IDO1 within the cytoplasm and cell membranes in normal cervical tissues. The prevalence of IDO1-positive cells was greater in cervical cancer specimens than in healthy cervical tissue. Furthermore, the expression levels of IDO1 in cancerous tissues were markedly higher compared to those in normal tissues. As in normal tissue, IDO1 was localized in the cytoplasm and membrane of cells in cervical cancer tissues (medium-intensity staining) (Fig. 1E and F).

### 3.2. Confirmation of high IDO1 expression in cervical cancer and its clinical relevance

Our observations revealed a greater presence of IDO1 in cervical cancer tissues compared to normal tissues. We performed Kaplan–Meier survival analysis using the clinical data obtained from TCGA to determine the clinical relevance of IDO1 expression in cervical cancer.

**Figure 2.**
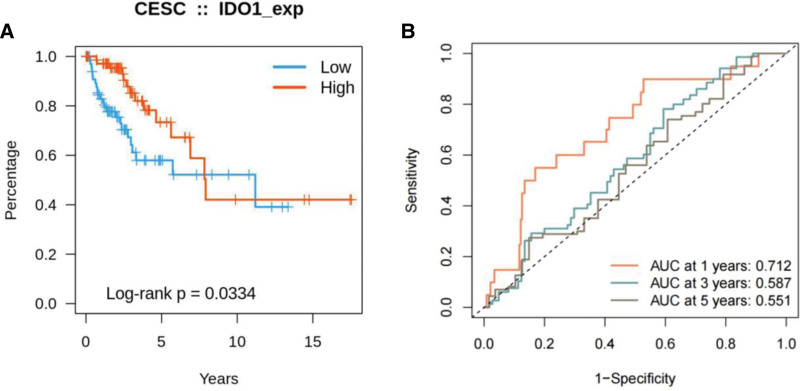
Validation of the prognostic and diagnostic values of IDO1 in CC. (A) Survival curve of patients with high or low IDO1 expression in cervical cancer. (B) Analysis of the relationship between IDO1 and overall survival in patients with cervical cancer

According to IDO1 expression levels in tissues, patients were classified into high- and low-expression groups. The study showed that patients with higher IDO1 expression levels had reduced OS rates in contrast to those with lower IDO1 expression, suggesting an adverse prognostic correlation (*P* = .034; Fig. 2A). For the patient survival rates at 1, 3, and 5 years, the AUCs of the high-expression group stood at 0.712, 0.587, and 0.551, in that order (Fig. 2B). Consequently, we ascertained that IDO1 expression most significantly influences the one-year survival rate and prognosis in cervical cancer patients. These findings suggested that IDO1 is an accurate prognostic marker for cervical cancer.

### 3.3. Relationship between IDO1 and immune cell infiltration in cervical cancer

**Figure 3.**
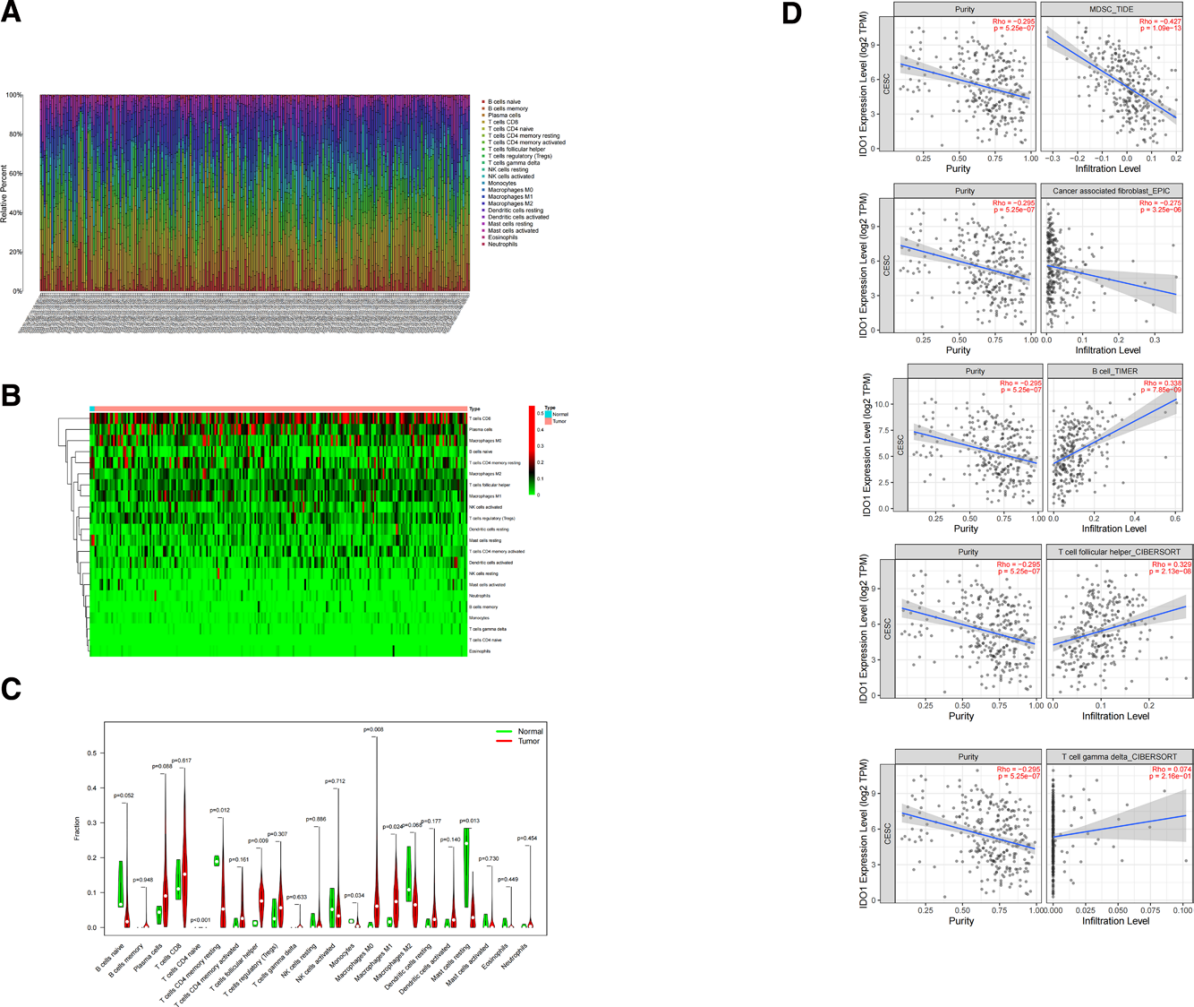
Correlation analysis of IDO1 expression and immune cell distribution. (A) Infiltration of various immune cells associated with cervical cancer. (B) Invasion of immune cells. (C) Component analysis of immune cells in cervical cancer and normal cervical tissues. (D) Correlation analysis of IDO1 expression and immune cell distribution.

Immune cell infiltration was closely associated with IDO1-predicted outcomes in TCGA data (Fig. 3A). Utilizing the CIBERSORT, vioplot, and pheatmap packages in R (3.4.1), we studied the infiltration of various immune cells in cervical cancer. A positive correlation between CD8 + T-cells and plasma cells was noted (Fig. 3B).

Further, various immune cell types, including CD8 + T-cells, plasma cells, follicular helper T-cells, Tregs, M0 macrophages, M1 macrophages, M2 macrophages, and others, showed notable infiltration in cervical cancer tissues compared to in healthy tissues (*P* = .032). Concurrently, there was a notable increase in the count of mast cells in the control group (*P* = .005) (Fig. 3C).

The intricate metabolic reprogramming within the tumor microenvironment profoundly influences treatment outcomes and patient survival.^[11]^ We observed that Myeloid Derived Suppressor Cells and cancer Associated Fib were negatively correlated with IDO1 expression. And we observed that B cell, T cell Follicular Helper and T cell Gamma Delta, positively correlated with IDO1 expression (Fig. 3D).

### 3.4. Correlation of IDO1 with immunoregulatory factors

**Figure 4.**
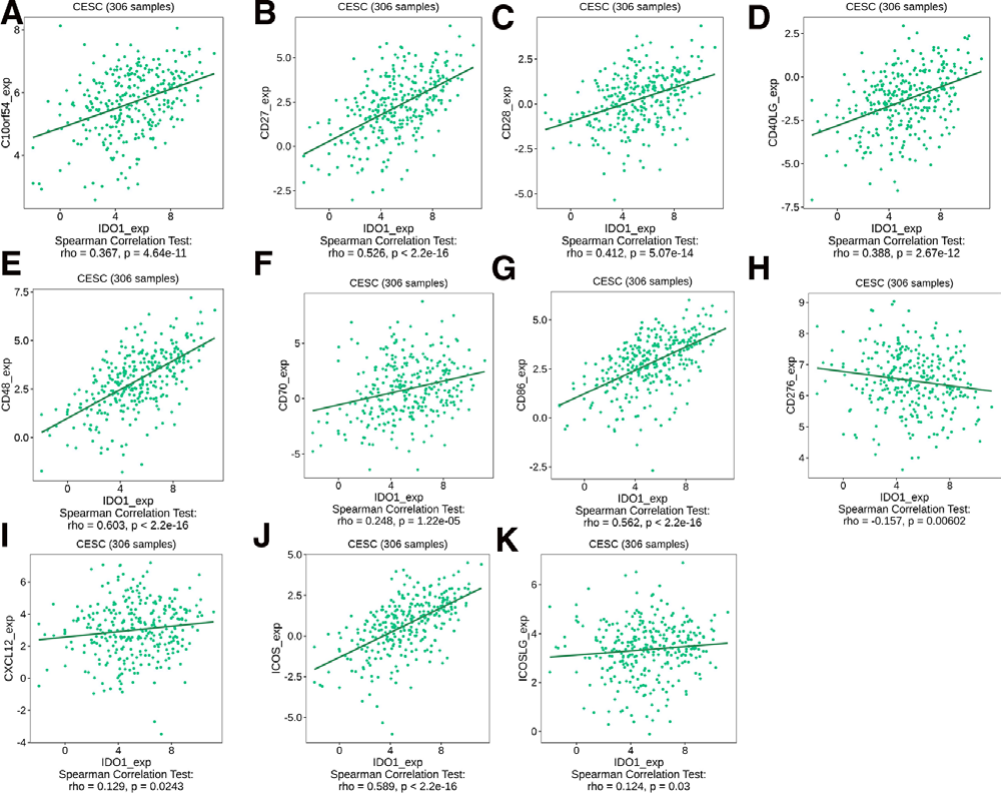
Differential analysis of tumor microenvironment scores based on IDO1 and immunomodulators: (A) c10orf54, (B) CD27, (C) CD28, (D) CD40LG, (E) CD48, (F) CD70, (G) CD86, (F) CD276, (I) CXCL12, (J) ICOS, and (K) ICOSLG.

We identified immune factors associated with IDO1 expression based on TISIDB data. Among immunostimulatory factors ICOS (ρ = 0.589), C10orf54 (ρ = 0.367), CD27 (ρ = 0.526), CD28 (ρ = 0.412), CD40LG (ρ = 0.388), CD48 (ρ = 0.603), CD70 (ρ = 0.248), CD86 (ρ = 0.562), CD276 (ρ = ‐0.157), CXCL12 (ρ = 0.129), and ICOSLG (ρ = 0.124), we observed a negative correlation with CD276, while all others were positively correlated with IDO1 expression (Fig. 4A–K).

**Figure 5.**
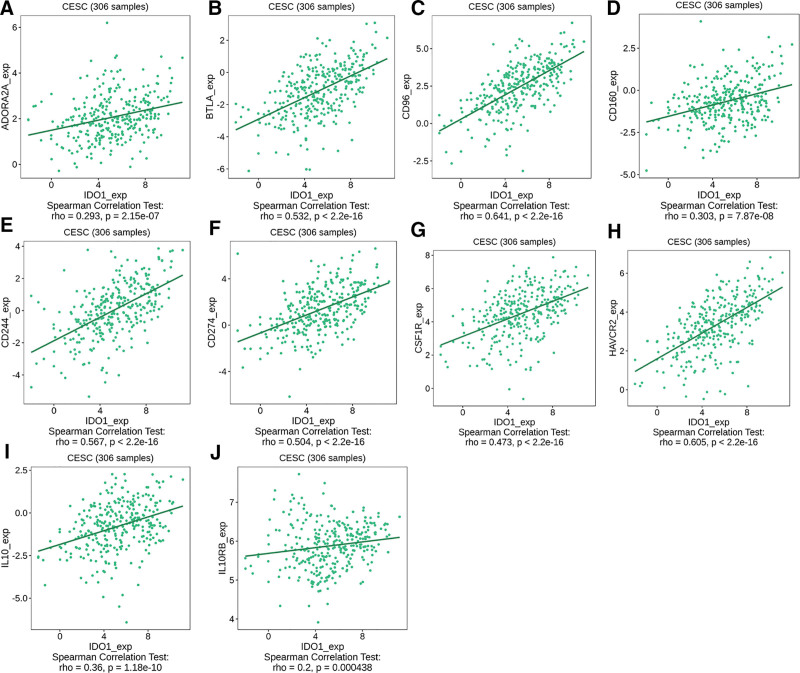
Interaction and functional analysis of immunoinhibitory and immunostimulatory factors: (A) ADORA2A, (B) BTLA, (C) CD96, (D)CD160, (E) CD244, (F) CD274, (G) CSF1R, (H)HAVCR2, (I) IL10, and (J) IL10RB.

The inhibitory factors ADORA2A (ρ = 0.293), BTLA (ρ = 0.532), CD96 (ρ = 0.641), CD244 (ρ = 0.567), CD160 (ρ = 0.303), CSF1R (ρ = 0.473), HAVCR2 (ρ = 0.605), IL10 (ρ = 0.36), and IL10RB (ρ = 0.2) exhibited a positive correlation with IDO1 expression (Fig. 5A–J).

### 3.5. Protein network and enrichment analysis of IDO1-related immune genes

**Figure 6.**
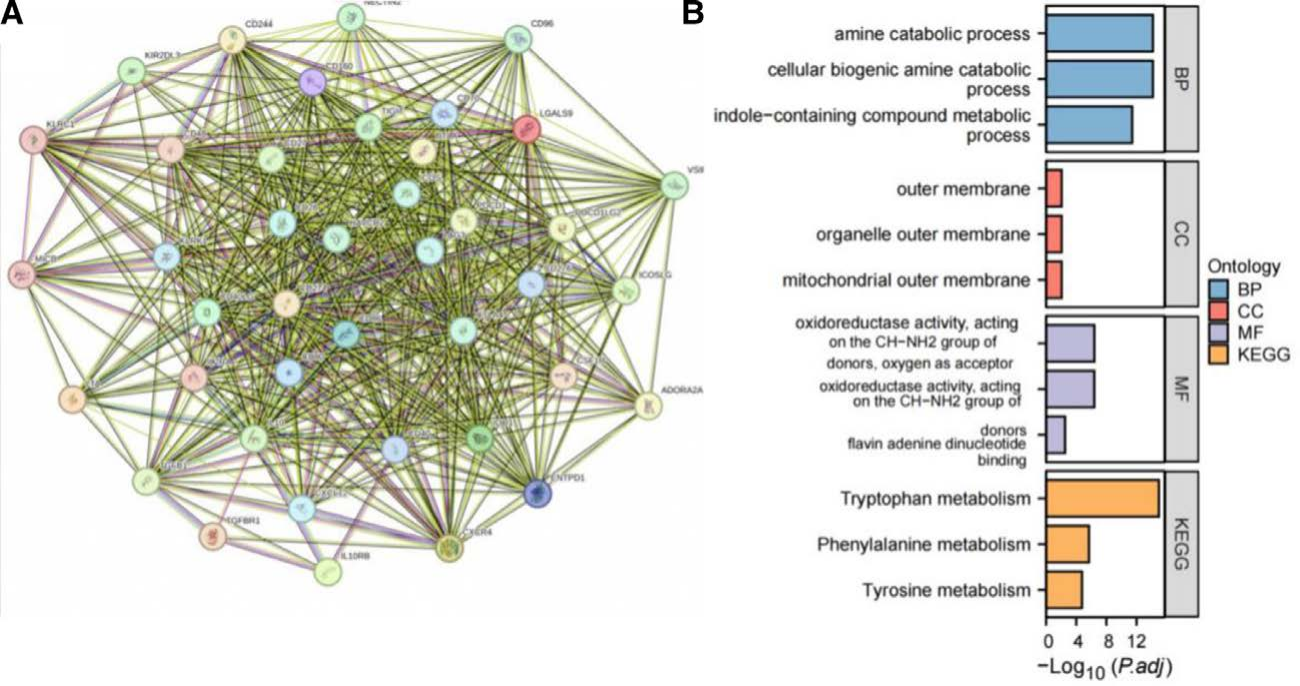
Interaction and functional analysis of immunoinhibitory and immunostimulatory factors. (A) PPI network of interactions between immunoinhibitory and immunostimulatory factors associated with IDO1. (B) GO and KEGG enrichment analyses for immunoinhibitory and immunostimulatory factors associated with IDO1.

Using the STRING website, we constructed a PPI network consisting of 9 immunosuppressive and 11 immunostimulatory agents, which included 41 nodes with 580 edges (Fig. 6A). GO enrichment analysis showed that most of these genes were associated with amine breakdown, cellular biological catabolism, metabolism of indole-containing compounds, and redox activity. KEGG analysis confirmed that IDO1 was closely related to tryptophan, phenylalanine, and tyrosine metabolism (Fig. 6B). We further dissected the association between IDO1 expression and immunomodulators to better understand the effect of IDO1 expression on tumor immunity.

### 3.6. Confirming the link between genes related to immunity and expression of IDO1 in cervical cancer patients

**Figure 7.**
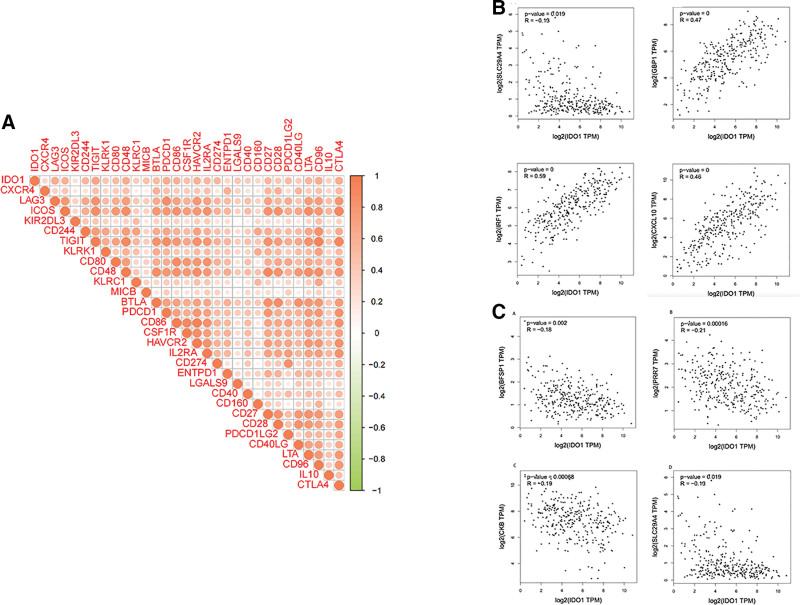
Interaction of immune factors with IDO1. (A) Association with IDO1-related immune genes. (B) A gene positively associated with IDO1 expression. (C) A gene negatively associated with IDO1 expression

Our univariate Cox regression analysis revealed a connection between 9 immunosuppressive and eleven immunostimulatory factors and cervical cancer prognosis. The hazard ratio values of TGFB1 and TGFBR1 were both > 1, indicating these as high-risk genes in cervical cancer. BTLA, CD96, LGALS9, CD28, CD80, CD48, KLRK1, ICOS, CD86, CD27, TIGHT, PDCD1, LAG3, HAVCR2, CTLA4, and CD244 had an hazard ratio < 1 and were thus considered low-risk factors (Fig. 7A). In addition, SLC29A4, GBP1, IRF1, and CXCL10 are positively correlated with IDO1, while BFSP1, PRR7, CKB, and SLC29A4 are negatively correlated with IDO1 (Fig. 7B and C). These results show a significant relationship between immune genes and target gene.

The construction of our risk score model was carried out in the following manner:

Risk score = (ExprCD96 × 2.03) − (ExprLGALS9 × 0.7) − (ExprTIGIT × 0.5) − (ExprKLRK1 × 0.45), where Exprrepr indicates the level of expression of each gene.

**Figure 8.**
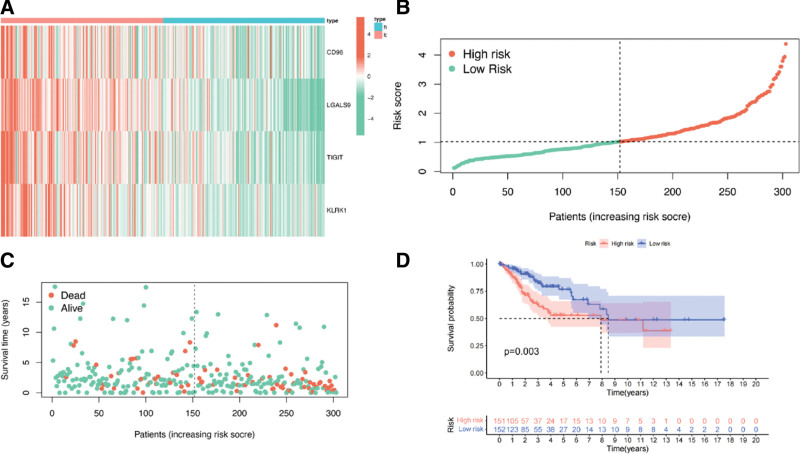
Establishment of the prognostic model of cervical cancer. (A) Risk score of IDO1 in patients with cervical cancer; a map of the risk profile based on IDO1-associated immune genes. (B) IDO1 risk score and immune factor-associated risk profile of patients with cervical cancer classified into low- and high-risk groups. (C) Survival of patients with cervical cancer with different risk scores. (D) Survival status map of immune genes associated with IDO1; survival analysis of patients with high- and low-expression of immune-related genes associated with IDO1.

Employing this equation, we determined the risk assessment for individuals diagnosed with cervical cancer. Based on the obtained risk score, we categorized patients into high- and low-risk groups. The risk distribution map, survival status map, and heat map of the representative immune genes related to IDO1 expression in TCGA are presented. We observed a negative correlation between the expression levels of CD96, LGALS9, TIGHT, KLRK1, and the risk score (Fig. 8A–C).

Our analysis of patient survival revealed a notable disparity between the low- and high-risk groups (*P* = .003). In particular, individuals classified as high-risk had a reduced lifespan compared to those in the low-risk category (Fig. 8D).

**Figure 9.**
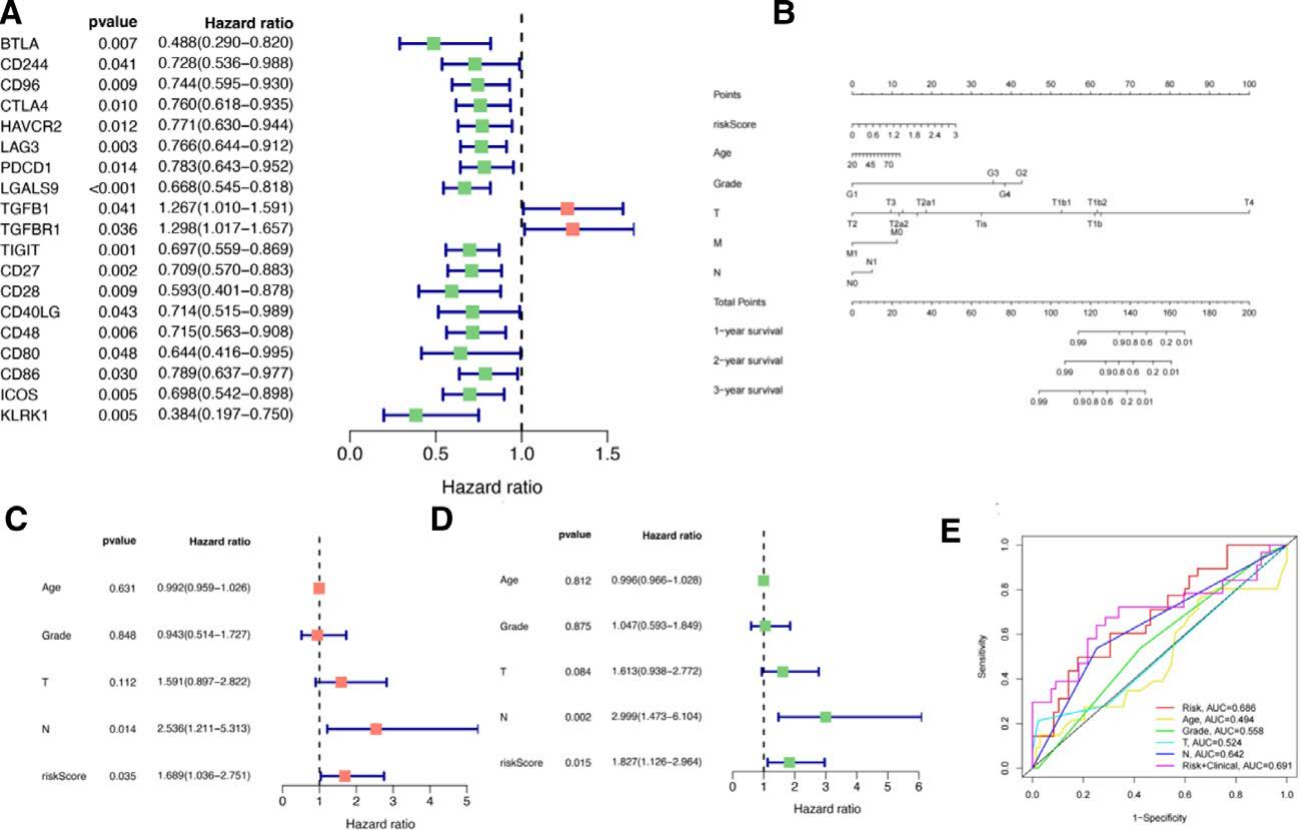
Prognostic risk factors of cervical cancer. (A) IDO1 immunoprognosis-related genes; a forest map of the prognostic model was established. (B) Calibration curve shows the 3-year OS score predicted by the nomogram. (C and D) Univariate and multivariate Cox regression analyses of IDO1-related immune genes were conducted to identify the prognostic factors associated with cervical cancer. (E) IDO1 clinical risk score, prognostic AUC values, and survival and time-dependent ROC curves for 1-, 3-, and 5-year OS models

We established a nomogram to predict the prognosis of patients with cervical cancer. We included the risk score, age, grade, and stage, obtaining the predicted AUC value of the model. Compared with other models, the AUC of the nomogram was the highest, indicative of the greatest prognostic accuracy. We used the monogram to accurately predict the 1-, 2-, and 3-year OS of patients with cervical cancer (Fig. 9A and B).

Both univariate and multivariate Cox regression studies revealed that the N stage and risk score independently predicted patient outcomes (*P* < .05), in contrast to age, grade, and T stage, which showed no independent correlation with prognosis (Fig. 9C and D).

Univariate Cox regression analysis also highlighted the N stage and risk score as prognostic factors (*P* < .05). The N stage had the most significant impact on the survival of patients. In contrast, factors like age, tumor grade, and T stage showed no correlation with OS in cervical cancer patients.

The AUC value of the risk score was 0.686, while the AUC value of the risk score plus clinical information was 0.691. The AUC value of grade was 0.558, that of the T stage was 0.524, and that of the N stage was 0.642. The AUC value of age was 0.494, indicating that age was not a risk factor affecting the duration of survival (Fig. 9E).

### 3.7. Immune checkpoint inhibitors in cervical cancer therapy

**Figure 10.**
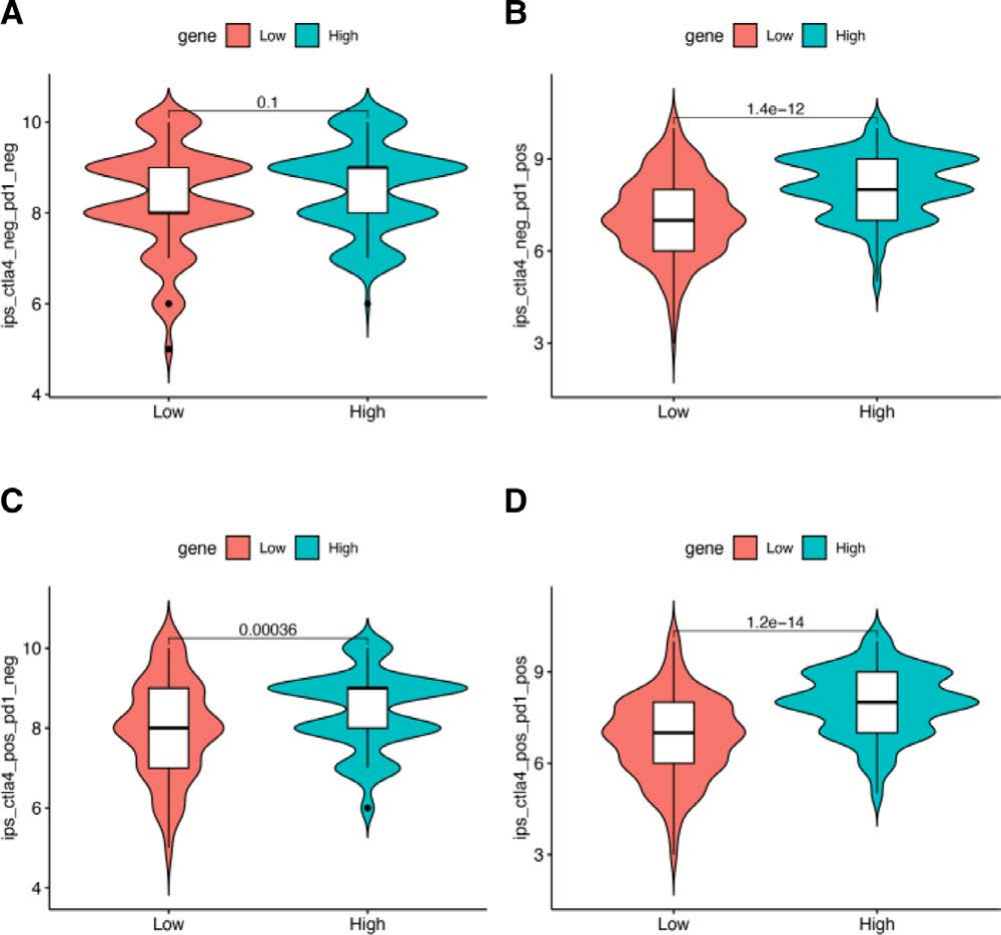
Immunophenotypic scores of patients with cervical cancer were downloaded from The Cancer Imaging Archive (TCIA) database to compare differences in IDO1 expression among the different immunotherapy groups.

In clinical settings, the key targets of immunotherapy are CTLA4 and PD-1/PD-L1, which significantly modulate the immune system by negatively regulating T-cell activation.^[12]^ Our analysis revealed a notable negative correlation between checkpoint inhibitor response and IDO1 expression, suggesting that immunotherapy is more effective in low-risk than in high-risk patients (Fig. 10A–D).

## 4. Discussion

Annually, over 500,000 women receive a cervical cancer diagnosis, leading to more than 300,000 fatalities globally.^[13]^ Extensive research has verified the role of immunomodulatory factors in cancer development, underscoring the crucial role of antitumor immunity in cancer progression.^[14,15]^

The tumor-immune microenvironment (TIME) is crucial in tumor-immune evasion, modulating tumor development and disease outcomes.^[16]^ Normally, cancer cells present antigens to DCs, which activate T-cells to identify and eliminate tumors by secreting granzyme and perforin. Tumor cells also employ immunosuppressive mechanisms such as the secretion of factors that cause immune cell dysfunction. As these facilitate immune escape, it is particularly important to study the immune microenvironment.^[17,18]^

An increasing number of immunotherapies are in development. The immunomodulatory enzyme IDO1 inhibits the function, proliferation, and activation of T-cells, thus impeding T-cell-dependent immune responses.^[19,20]^ Excessive IDO1 expression in tumors leads to a reduction in Trp and an increase in Kyn metabolites, creating a tumor microenvironment that suppresses the immune system and enables tumor-immune evasion. Additionally, IDO1 upregulation is linked to unfavorable outcomes in different cancer types.^[21]^ Studies have explored the interaction between cervical cancer and IDO1-related immunosuppression as well as the interaction between target genes and cervical cancer-related immune cells, reporting a new prognostic model for cervical cancer.^[20]^

In the course of tumor growth, a range of cells are drawn to the TIME. These cells inhibit the infiltration and function of T-cells to suppress antitumor immunity. Compared with that in the healthy cervix, the expression of IDO1 is significantly enhanced in cervical cancer, negatively correlating with immune cell infiltration and influencing prognosis. There is a notable inverse correlation between IDO1 expression and various immune cell types, such as CD8 + T-cells, NK cells, and Tregs. Recent studies show a strong link between the substantial presence of CD8 + T-cells and extended survival in clear cell renal cell carcinoma and prostate cancer.^[14]^ Furthermore, NK cells can be activated to recognize and kill tumor cells through a set of stimulatory and inhibitory receptors, thus potentially amplifying immune responses.^[22]^ The observed inverse relationship between IDO1 expression and NK cells in cervical cancer could be a significant factor in the integrated approach to treating cervical cancer.

The current study focused on IDO1 in the context of tumor immunology. TISIDB was employed to examine immune infiltration in cervical cancer, revealing that IDO1 levels exhibit a significantly positive association with ICOS, C10orf54, CD27, CD28, CD70, and other immunostimulatory factors, which negatively regulate the expression of CD276. CD27 and CD28, are involved in cell cycle regulation.^[23]^ Simultaneous stimulation of CD27/CD70 has the potential to stimulate T-cells, boosting their transformation into cytotoxic and memory T-cells. T-cells that produce CD27 and CD70 are crucial in controlling the activation of B-cells and the production of immunoglobulins. The role of CD27 signaling in cancer can thus be exploited for antitumor therapy.^[24]^ Among the immunoinhibitory factors associated with IDO1, CD96 and CDHAVCR2 were positively correlated with the expression of IDO1. CD96 represents a new target for immune checkpoint receptors, known to either enhance or suppress NK cell activation, thus influencing the adhesion and spread of tumor cells.^[25]^ Moreover, CD96 was reported to inhibit NK cell-induced cytokine responses and control cytokine release in Th9 cells. We found that the roles of CD96 and IDO1 are closely related, suggesting that tumor-immune regulation by IDO1 could be of prognostic relevance in cervical cancer.^[26]^ These results suggested that IDO1 may influence the therapeutic response in cervical cancer via immune checkpoint regulation.

The expression of BTLA, KLRK1, CD80, and CD28 reduced the risk score, and these were suggested as protective factors in cervical cancer prognosis. TGFB1 and TGFBR1 are known as high-risk factors for cervical cancer. TGFB1 is a strong growth suppressor and is considered to have tumor-suppressive properties, regulating cell growth, differentiation, and proliferation.^[27]^ Additional research indicates that TGFB1 indirectly triggers PIK3CA/AKT1 signaling, which, in turn, increases the spread of prostate cancer cells.^[28]^ Additionally, TGFBR1 is considered a tumor susceptibility allele with high occurrence and low penetration in various cancers, including gastric and breast cancer, elevating the likelihood of cancer development.^[29]^ BTLA can inhibit anticancer immunity, and BTLA polymorphisms were found to increase susceptibility to various cancers. For example, the expression of BTLA is upregulated in gallbladder cancer and elevated in the T-cells of patients with melanoma and lung cancer.^[30]^

Effective treatment for cervical cancer can be achieved by utilizing targeted therapy at an early stage, combined with appropriate chemotherapy drugs, which can stimulate the immune system of the host.^[31]^ Elevated levels of TGFB1 and TGFBR1 in cervical cancer patients were associated with a lower OS, while BTLA, KLRK1, CD80, and CD28 expression had an inverse relationship with OS. Analysis at the prognostic, univariate, and multivariate levels revealed the potential of the risk score as a standalone prognostic indicator in cervical cancer patients.

PD-L1 and CTLA4 are key prognostic biomarkers for neoadjuvant chemoradiotherapy in advanced tumors. Anti-CTLA4 is thought to be one of the best predictors of melanoma response. In Merkel cell carcinoma, clinical responses are associated with cells that highly express PD1 and PD-L1, with the former being expressed in a variety of TME-associated immune cells.^[14]^ Antibodies targeting PD1 or PD-L1 are capable of reinstating T-cell activity to trigger an immune reaction against cancerous cells. Various combined treatments, such as anti-PD1/PD-L1 combined with angiogenesis inhibitors, targeted therapies, other immune checkpoint inhibitors, or metabolic modulators, are proven to be more effective against cancer than monotherapy.^[32]^

The present study did have certain limitations, as analysis was solely based on TCGA data. Additional experimental and clinical confirmation are thus warranted.

## 5. Conclusions

In the present study, IDO1 expression correlated with unfavorable outcomes and immune infiltration in cervical cancer patients. By investigating immune genes linked to IDO1, we developed a predictive model for risk scores. Overall, IDO1 holds potential as a therapeutic target for cervical cancer.

## Author contributions

**Conceptualization:** Cong Xu.

**Data curation:** Cong Xu, Min Wang, Yonghong Xu.

**Formal analysis:** Cong Xu.

**Investigation:** Cong Xu.

**Software:** Cong Xu, Chaowen Chen, Fang Liu.

**Supervision:** Cong Xu.

**Visualization:** Cong Xu, Guangming Wang.

**Writing – original draft:** Cong Xu.

**Writing – review & editing:** Guangming Wang.
